# Sivelestat-Loaded Neutrophil-Membrane-Coated Antioxidative Nanoparticles for Targeted Endothelial Protection in Sepsis

**DOI:** 10.3390/pharmaceutics17060766

**Published:** 2025-06-10

**Authors:** Juexian Wei, Aijia Zhong, Yuting Zhang, Ehua Deng, Hengzong Mo, Hongyu Zhao, Jiayu Huang, Huaidong Peng, Kaiyin Zhang, Xiaohui Chen, Haifeng Mao, Yixin Chen, Yongcheng Zhu

**Affiliations:** 1 Department of Emergency, The Second Affiliated Hospital, Guangzhou Medical University, Guangzhou 510260, China; wofengchulong@126.com (J.W.); 13786201605@163.com (H.Z.); jiayuhuang1@stu.gzhmu.edu.cn (J.H.); kaiyin_zhang@163.com (K.Z.); cxhgz168paper@163.com (X.C.); 2017682033@gzhmu.edu.cn (H.M.); 2 School of Pharmaceutical Sciences, Guangzhou Medical University, Guangzhou 511436, China; 2022123058@stu.gzhmu.edu.cn (A.Z.); 2023118116@stu.gzhmu.edu.cn (E.D.); 3 Department of Physiology, School of Basic Medical Sciences, Guangzhou Medical University, Guangzhou 511436, China; zytingzy@163.com; 4 Department of Clinical Medicine, The Second Clinical Medicine School of Guangzhou Medical University, Guangzhou 510260, China; 2021111284@stu.gzhmu.edu.cn; 5 Department of Pharmacy, The Second Affiliated Hospital, Guangzhou Medical University, Guangzhou 510260, China; phdloveyou@163.com

**Keywords:** neutrophil-membrane-coated nanoparticles, sivelestat, endothelial injury, sepsis, ROS scavenging, drug delivery system

## Abstract

**Background/Objectives**: This study aims to develop and evaluate neutrophil-membrane-coated nanoparticles (Siv@NMs) encapsulating sivelestat for the treatment of sepsis-induced endothelial injury. Leveraging the intrinsic chemotactic properties of neutrophil membranes, Siv@NMs are engineered to achieve site-specific delivery of sivelestat to damaged endothelia, thereby overcoming the limitations of conventional therapies in mitigating endothelial dysfunction and multiorgan failure associated with sepsis. **Methods**: Siv@NMs were synthesized through a combination of ultrasonication and extrusion techniques to encapsulate sivelestat within neutrophil-membrane-derived vesicles. Comprehensive physicochemical characterization included analysis of particle size distribution, zeta potential, and encapsulation efficiency. Stability profiles and controlled release kinetics were systematically evaluated under simulated conditions. In vitro investigations encompassed (1) endothelial cell biocompatibility assessment via cytotoxicity assays, (2) investigation of the targeting efficiency in suppressing endothelial neutrophil extracellular trap generation during inflammation, and (3) ROS-scavenging capacity quantification using flow cytometry with DCFH-DA fluorescent probes. In vivo therapeutic efficacy was validated using a cecal ligation and puncture (CLP) sepsis mouse model, with multiparametric monitoring of endothelial function, inflammatory markers, ROS levels, and survival outcomes. **Results**: The optimized Siv@NMs exhibited an average particle size of approximately 150 nm, and a zeta potential of −10 mV was achieved. Cellular studies revealed that (1) Siv@NMs selectively bound to inflammatory endothelial cells with minimal cytotoxicity, and (2) Siv@NMs significantly reduced ROS accumulation in endothelial cells subjected to septic stimuli. In vitro experiments demonstrated that Siv@NMs treatment markedly attenuated endothelial injury biomarkers’ expression (ICAM-1 and iNOS), suppressed formation of neutrophil extracellular traps, and improved survival rates compared to treatment with free sivelestat. **Conclusions**: The neutrophil-membrane-coated nanoparticles loaded with sivelestat present a breakthrough strategy for precision therapy of sepsis-associated endothelial injury. This bioengineered system synergistically combines targeted drug delivery with multimodal therapeutic effects, including ROS mitigation, anti-inflammatory action, and endothelial protection. These findings substantiate the clinical translation potential of Siv@NMs as a next-generation nanotherapeutic for sepsis management.

## 1. Introduction

Sepsis is a life-threatening severe inflammatory response syndrome that leads to multiorgan dysfunction and dysregulated host immunity, significantly increasing the mortality rate [1]. Endothelial injury represents a central pathological mechanism driving organ failure in sepsis [2,3,4]. The resulting vascular hyperpermeability not only exacerbates disease progression but also serves as a critical gateway to widespread organ damage [5,6]. Consequently, restoring endothelial function has emerged as a key therapeutic strategy for sepsis treatment [7]. Within the pathophysiological cascade of sepsis, maintaining vascular endothelial integrity and functionality is paramount, as immune-mediated dysfunction fundamentally contributes to sepsis-induced organ failure.

Sivelestat, a selective neutrophil elastase inhibitor, has demonstrated therapeutic efficacy in acute respiratory distress syndrome (ARDS) by reducing alveolar damage [8] and suppressing neutrophil extracellular trap (NET) formation [9,10], processes intimately linked to endothelial dysfunction in sepsis [11]. Through inhibition of excessive NE activity, sivelestat helps maintain vascular integrity and attenuate inflammatory endothelial injury [12]. However, its clinical application is limited by a short plasma half-life, necessitating frequent administrations and imposing substantial treatment costs for patients [13,14]. Therefore, improving its delivery efficiency and extending its therapeutic duration are crucial. Targeted delivery of sivelestat to injured endothelial cells in sepsis could enhance its localized protective effects while offering a more cost-effective treatment approach.

Neutrophil-membrane-coated nanoparticles and neutrophil-derived liposomes have recently gained attention as promising biomimetic carriers for drug delivery [15,16], capitalizing the innate ability to home to sites of inflammation [17]. By preserving key surface markers, such as LFA-1 and CD11b, these nanoparticles can selectively interact with immune cells and inflamed endothelium, promoting site-specific accumulation [18]. Moreover, neutrophil membranes inherently exhibit anti-inflammatory properties, partly due to phosphatidylserine externalization during apoptosis. This surface-exposed phosphatidylserine facilitates macrophage recognition and phagocytosis of apoptotic neutrophils, driving a shift from pro-inflammatory to anti-inflammatory macrophage phenotypes that aids inflammation resolution [19]. ROS are recognized as key drivers of endothelial injury and multiorgan dysfunction in sepsis. Recent work describes a range of antioxidative nanotherapies that quench ROS and restore redox balance [20]. Ceria-based particles [21] and membrane-camouflaged vesicles loaded with antioxidant drugs [22] have each shown the ability to lower oxidative stress. Neutrophil-mimetic carriers that combine ROS-scavenging capacity with inflammatory site homing further reduce tissue damage in CLP and LPS models, demonstrating that targeted delivery and antioxidative action can work synergistically to improve therapeutic outcomes [23]. Consequently, these intrinsic immunomodulatory properties enable neutrophil membrane-based nanoparticles to mitigate excessive immune responses, further enhancing their therapeutic potential in inflammatory diseases [22]. Owing to these immune-evasive capabilities and targeted delivery properties, neutrophil-membrane-coated nanoparticles have been extensively explored for tumor-targeted therapies and are increasingly investigated for antioxidative drug delivery in immune-related disorders.

To overcome these limitations, we developed neutrophil-membrane-encapsulated sivelestat nanoparticles (Siv@NMs) for targeted delivery to injured endothelial cells under septic conditions. This strategy is expected to improve drug bioavailability while reducing treatment duration. Given that immune-mediated endothelial dysfunction is a key driver of sepsis-induced organ failure—with oxidative stress playing a central role in endothelial injury [24]—we conducted a series of experiments to evaluate (i) the effects of Siv@NMs on sepsis-induced renal dysfunction and NETs formation, and (ii) their ability to enhance endothelial barrier integrity and reduce ROS generation in LPS-stimulated endothelial cells. These investigations were designed to assess the therapeutic potential of Siv@NMs as a targeted nanoplatform for treating sepsis-induced endothelial injury.

## 2. Materials and Methods

### 2.1. Materials, Cells, and Animals

Sivelestat with 99% purity was purchased from Sigma-Aldrich (CAS No. 127373-66-4, St. Louis, MO, USA). All other required materials, solvents, and chemicals were purchased from commercial sources and used upon receipt. Male C57BL/6 mice, aged 4 weeks and weighing between 15 and 20 g, were acquired from the Guangdong Laboratory Animal Center in Guangzhou, China. The mice were kept under standard housing conditions and provided with a regular laboratory diet. All animal tests were approved by the GMU research ethics committee (No. GY2023-336). HL-60 cells (Pricella, Wuhan, China) were cultured in Iscove’s Modified Dulbecco’s Medium (IMDM) with 10% fetal bovine serum (FBS) and 1% penicillin-streptomycin (P/S) at 37 °C in a 5% CO_2_ humidified incubator. Human umbilical vein endothelial cells (HUVECs) were cultured in endothelial cell growth medium (ECGM) supplemented with 5% fetal bovine serum (FBS) and 1% penicillin-streptomycin (P/S) at 37 °C in a humidified incubator with 5% CO₂. HUVECs (1 × 10^6^ cells/mL) were seeded in 6-well plates and divided into four groups: Control, LPS (1 μg/mL LPS for 24 h), LPS+Siv (1 μM sivelestat + 1 μg/mL LPS for 24 h), and LPS+Siv@NMs (1 μM sivelestat + 1 μg/mL LPS for 24 h). Cells were pre-treated with 1 μg/mL of LPS for 2 h, followed by the addition of 1 μM of sivelestat, and then incubated for an additional 22 h, totaling 24 h of treatment. Importantly, quantitative analysis confirmed that the final concentration of sivelestat in both the LPS+Siv and LPS+Siv@NMs groups was maintained at 1 μM, ensuring consistent dosing across these treatment conditions.

### 2.2. Animal Model

Cecal ligation and puncture (CLP) was used to induce sepsis in mice, as described in a previous study [25]. Thirty minutes before the procedure, the mice received subcutaneous administrations of buprenorphine (0.1 mg/kg; Buprecare^®^, Axience, Pantin, France) for pain relief. The CLP procedure, lasting approximately 10 min, was performed under general anesthesia induced with 3.5% isoflurane in an inhalation chamber and maintained with 1.5% isoflurane and 98.5% oxygen throughout the surgery. Following fur removal and sterilization, a median cut was made in the abdomen to expose the cecum. The contents of the cecum were gently displaced toward its distal end, and a portion of the cecal base was ligated. The cecum was perforated using a 21G needle, allowing some fecal matter to extrude. The cecum was then repositioned into the abdominal cavity, and the skin was sutured in a layered fashion. In the sham group, the cecum was exposed but neither ligated nor perforated. Animals were rehydrated 0.5 h before surgery, subjected to CLP at time 0, then administered an intravenous injection of sivelestat or Siv@NMs at 1 h post-surgery. Four experimental groups were established: Sham, CLP, CLP+Siv, and CLP+Siv@NMs. In the CLP+Siv group, sivelestat was administered at a dose of 10 mg/kg via intravenous injection, while in the CLP+Siv@NMs group, the injected dose was equivalent to 10 mg/kg of sivelestat. Based on dosing regimens reported in various studies, and to verify that Siv@NMs can exert therapeutic effects at a lower dose, we additionally established a lower-concentration group within the CLP+Siv cohort [26,27]. To evaluate survival, additional injections were given every 24 h until 60 h post-surgery. At 24 h, organ and blood samples were harvested for further analyses.

### 2.3. Extraction of the Neutrophil Membrane

HL-60 cells were obtained according to previously described methods [28]. To differentiate into neutrophil-like cells (dHL-60), HL-60 cells were switched to RPMI-1640 medium with 10% FBS, 1% P/S, and 1.2% DMSO for 4 days. After that, the neutrophil membrane (NM) was isolated from the stimulated cells. Briefly, neutrophils were treated with the hypotonic lysing buffer supplemented with 1× protease inhibitors and then underwent ultrasonication to promote cell lysis. Subsequently, the suspension was centrifuged (2000× *g*, 5 min) at 4 °C to remove unbroken nuclei and cells. The resulting supernatant was centrifuged again (13,000× *g*, 45 min) to obtain high-purity NM.

### 2.4. Preparation of Siv@NMLs

Firstly, sivelestat was dissolved in dimethyl sulfoxide (DMSO) to prepare a 40 μM stock solution. Briefly, 18 μL of the stock solution was added to 2 mL of ultrapure water, followed by continuous stirring under light-protected conditions for 13 h. After that, the resulting drug solution was filtered through an 0.8 μm micropore membrane and thoroughly mixed with the extracted neutrophil membrane (NM). Subsequently, the mixture was subjected to ultrasonication to achieve drug loading, yielding Siv@NMs.

### 2.5. Characterization of Siv@NMs

A dynamic light scattering (DLS) system was utilized to evaluate the physicochemical properties of Siv@NMs, including their particle size distribution, polydispersity index (PDI), and zeta potential. The morphologies of Siv@NMs were observed using a transmission electron microscope (TEM). Siv@NMs were suspended in phosphate-buffered saline (PBS) supplemented with various concentrations of fetal bovine serum (FBS), and changes in both particle size and PDI were monitored over a seven-day period to evaluate their stability.

### 2.6. Cell Proliferation Activity and Hemocompatibility of Siv@NMs

HUVECs were seeded into 96-well plates and cultured for 24 h before treatment. Subsequently, the cells were exposed to various concentrations (5–200 ng/mL) of blank neutrophil membranes (Blank NMs), free sivelestat (Siv), and Siv@NMs for an additional 24 h, after which cell viability was assessed for each group.

For the hemocompatibility assay, a 2% erythrocyte suspension was prepared and incubated at 37 °C for 2 h with test solutions, including Triton X-100 (positive control), 0.9% NaCl (negative control), Blank NMs, free sivelestat (Siv), and Siv@NMs (200 ng/mL). Following incubation, the samples were centrifuged at 1000 g for 5 min, and the supernatants were collected and analyzed using an ultraviolet spectrophotometer (Thermo Fisher Scientific, Waltham, MA, USA) at 576 nm to determine the absorbance values of each group. The hemolysis rate was subsequently calculated using the equation: hemolysis rate (%) = [(OD_samples_ − OD_negative control_)/(OD_positive control_ − OD_negative control_)] × 100%. The cytotoxicity of Blank NMs, Siv, and Siv@NMs in HUVECs was assessed using the CCK-8 assay (Beyotime Biotechnology, Shanghai, China), following the manufacturer’s instructions. Briefly, HUVECs were seeded in 96-well plates at a density of 10^4^ cells per well in 100 μL of medium. The cells were treated with varying concentrations of Blank NMs, Siv, or Siv@NMs (0, 5, 10, 20, 40, 100, and 200 μM) for 24 h. After the incubation, 10 μL of CCK-8 reagent was added to each well, and the plates were further incubated at 37 °C in a 5% CO_2_ atmosphere for 4 h. The absorbance at 450 nm was then recorded using a Tecan Infinite F200 microplate reader (Männedorf, Switzerland), and cell viability was calculated accordingly.

### 2.7. Fluorescent Observation of Siv@NMs

According to a previous study [29], we prepared fluorescence-labeled Siv@NMs by conjugating them with Dil. HUVECs, pre-stimulated with LPS for 12 h, were then stained with DAPI and incubated with the Dil-labeled Siv@NMs for 30 min, after which the cells were observed using a fluorescence microscope (Carl Zeiss, Oberkochen, Germany).

### 2.8. H&E Staining

Tissue sections from mouse kidneys were deparaffinized and rehydrated by immersing them in xylene I and II for 10 min each, followed by a series of ethanol solutions (100%, 95%, 85%, and 75%) for 5 min each. The sections were then rinsed in distilled water for 5 min. Next, the sections were stained with hematoxylin solution for 5–10 min and rinsed in running tap water for 5 min. Differentiation was achieved by immersing the sections in 1% acid alcohol for a few seconds, followed by rinsing in running tap water for 5 min. The sections were then blued in 0.2% ammonia water for 1 min and rinsed again in running tap water for 5 min. Subsequently, the sections were stained with eosin solution for 1–2 min and quickly rinsed in distilled water to remove excess stain. Dehydration was performed through a series of ethanol solutions (75%, 85%, 95%, and 100%) for 5 min each. The sections were cleared in xylene I and II for 5 min each. Finally, the sections were mounted with a suitable mounting medium and coverslipped. The stained sections were observed under a light microscope, and images were captured as needed.

### 2.9. TUNEL Assay

Following the deparaffinization and rehydration steps described in the H&E staining procedure, sections were treated with 20 µg/mL of Proteinase K (without DNase) at 20–37 °C for 15–30 min. The sections were then thoroughly washed with PBS or HBSS three times to remove any residual Proteinase K. After washing, sections were incubated in 3% hydrogen peroxide solution in PBS at room temperature for 20 min to block endogenous peroxidase activity, followed by three washes in PBS. The TUNEL reaction mixture was prepared and applied, and sections were incubated at 37 °C for 1 h in the dark. After washing with PBS, sections were incubated with HRP-conjugated secondary antibody for 30 min, followed by DAB substrate until the desired stain developed. Finally, the sections were counterstained with hematoxylin, dehydrated through graded ethanol, cleared in xylene, and mounted. Images were captured using a light microscope.

### 2.10. JC-1 Assay

Reagents for the JC-1 assay were purchased from Biyuntian Biotechnology (Shanghai, China). HUVECs were seeded in 96-well plates and treated under the experimental conditions. Following treatment, the cells were washed with PBS and incubated with JC-1 dye (typically 5 µM in serum-free medium) at 37 °C in the dark for 20–30 min. After incubation, the fluorescence intensities of the JC-1 monomers (green) and aggregates (red) were measured using a microplate reader with appropriate excitation and emission settings. The red/green fluorescence ratio was then calculated to assess changes in mitochondrial membrane potential, where a decrease in this ratio indicates mitochondrial depolarization.

### 2.11. ROS Detection

Reagents for the ROS detection assay were purchased from Biyuntian Biotechnology (Shanghai, China). For the evaluation of intracellular ROS levels, HUVECs were cultured and treated as described above. Following the treatment period, the cells were incubated with a 10 µM solution of DCFH-DA for 30 min at 37 °C in the dark. After staining, cells were washed with cold PBS, harvested, and analyzed by flow cytometry. The fluorescence intensity, corresponding to the oxidation of DCFH to DCF by ROS, was recorded and quantified to determine the percentage of ROS-positive cells, providing an indication of oxidative stress levels under each treatment condition.

### 2.12. ELISA

Additionally, MPO-DNA and H3cit levels in plasma were measured using ELISA kits from Meimian (Meimian Biotechnology Co., Ltd., Nantong, China). TNF-α and IL-6 levels in plasma were measured using ELISA kits from Dakewe (Dakewe Biotech Co., Ltd., Shenzhen, China). Optical density (OD) values were read at 450 nm using a microplate reader within 5 min. Standard curves were generated for quantification. Both standards and samples were analyzed in triplicate.

### 2.13. Western Blotting

Western blot analysis was performed to identify the characteristic neutrophil markers on both NMs and Siv@NMs, following the protocol described previously [30]. In brief, total protein was extracted from cells using radioimmunoprecipitation assay buffer, which contained a mixture of protease inhibitors and a protein phosphatase inhibitor (Kaiji Company, Shenzhen, China). The extracted proteins were then separated via SDS-PAGE and transferred onto polyvinylidene fluoride (PVDF) membranes. The membranes were incubated overnight at 4 °C with primary antibodies targeting GADPH, TNF-α receptor (TNF-αR), CD66, iNOS, *p*-ERK, and ICAM-1 (all from Cell Signaling Technology Inc., Danvers, MA, USA), and CD11β and LAF-1 antibody were obtained from Abcam (Waltham, MA, USA). The next day, the membranes were incubated with the corresponding secondary antibodies. The expression levels of the target proteins were analyzed using ImageJ software (Version 1.38e, National Institutes of Healthre, Bethesda, MD, USA).

### 2.14. Statistics Analysis

The quantitative data are presented as mean ± SD. Statistical analysis was performed using GraphPad Prism 9.0 (San Diego, CA, USA). Group differences were assessed using one-way ANOVA followed by Tukey’s post hoc test for multiple comparisons. Owing to the small sample size and ordinal nature of some data, the Kruskal–Wallis H test was also employed, with Dunn’s post hoc test and Bonferroni correction applied to identify specific group differences. Kaplan–Meier survival curves were generated to analyze survival data, and the log-rank (Mantel–Cox) test was used to compare survival distributions among groups. A *p*-value < 0.05 was considered statistically significant.

## 3. Results

### 3.1. Characterization of Siv@NMs

**Figure 1:**
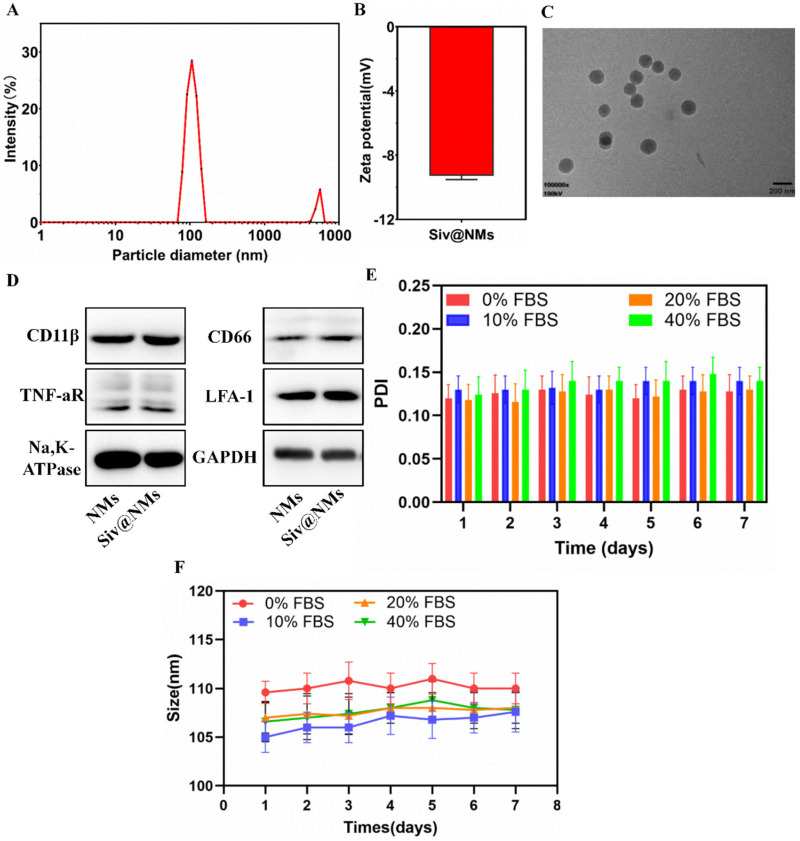
Characterization of Siv@NMs. (**A**) Particle diameter distribution analysis and (**B**) surface zeta potential measurements of Siv@NMs. (**C**) Typical morphology of Siv@NMs visualized by TEM, where each particle possesses a darker electron-dense core surrounded by a lighter, thin peripheral rim. (**D**) Western blot analysis showing the presence of specific neutrophil membrane marker proteins in neutrophil membranes (NMs) and Siv@NMs. (**E**,**F**) Stability assessment of Siv@NMs, indicated by changes in the particle dispersion index (PDI) and hydrodynamic diameter, incubated in PBS supplemented with different concentrations of fetal bovine serum (FBS) over 7 days. Data are expressed as the mean ± SD (n = 5).

The physicochemical properties of Siv@NMs were systematically characterized. Dynamic light scattering (DLS) characterization revealed that Siv@NMs displayed a largely monodisperse size distribution with excellent dispersibility. The particle size distribution was predominantly centered at around 105.9 nm (91.7% intensity), while a minor secondary peak was observed at nearly 5558.8 nm (8.3% intensity), with the calculated Z-average at 166.9 ± 18.3 nm (Figure 1A), indicating favorable colloidal dispersion. Zeta potential measurements revealed a negative charge surface of approximately −10 mV (Figure 1B), consistent with previously reported neutrophil-membrane-derived nanoparticles [31]. TEM further confirmed the spherical morphology and smooth surface topography of Siv@NMs, with particle dimensions concordant with DLS measurements. Each particle possesses a darker electron-dense core surrounded by a lighter, thin peripheral rim (Figure 1C). Western blot analysis verified the successful retention of key neutrophil membrane-specific proteins on Siv@NMs, including CD66, TNF-aR, CD11b, and LFA-1 (Figure 1D). The preservation of these signature biomarkers on Siv@NMs indicated effective preservation of neutrophil membrane integrity following the nanoparticle fabrication. To assess biological stability, Siv@NMs were incubated in PBS containing varying fetal bovine serum (FBS) concentrations (0–40%) over seven days. Remarkably, neither particle sizes nor PDI values of Siv@NMs exhibited significant alterations during this period, suggesting exceptional colloidal stability under physiologically relevant conditions.

### 3.2. Biocompatibility of Siv@NMs

**Figure 2:**
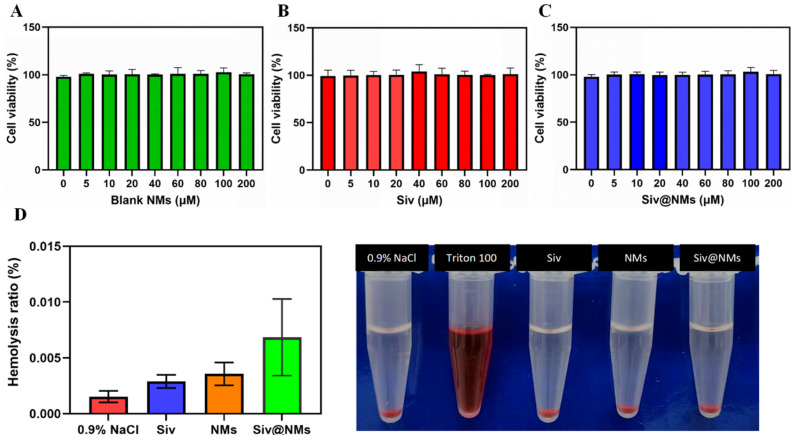
Cytocompatibility and hemocompatibility of Siv@NMs. The HUVECs’ viability after incubating with various concentrations of (**A**) Blank NMs, (**B**) sivelestat, (**C**) Siv@NMs, (**D**) hemolysis ratio (left), and hemocompatibility test (right). Data are expressed as the mean ± SD (n = 5).

The biocompatibility profile of Siv@NMs was systematically evaluated in human umbilical vein endothelial cells (HUVECs) using the CCK-8 assay. As shown in Figure 2A–C, HUVECs maintained high viability (>90%) following 24 h exposure to varying concentrations (0–200 μM) of blank neutrophil membranes (Blank NMs), free sivelestat (Siv), and Siv@NMs, demonstrating excellent cytocompatibility and minimal cytotoxic effects on endothelial cells. To further assess blood compatibility, the hemolysis assay was performed using blood samples collected from healthy rats. Quantitative analysis revealed negligible hemolytic activity (<0.01%) for all test groups, including 0.9% NaCl (negative control), free Siv, Blank NMs, and Siv@NMs (Figure 2D). This was in stark contrast to the positive control (Triton X-100), which induced significant hemolysis. Visual inspection of the samples further confirmed these quantitative findings, collectively indicating that Siv@NMs exhibit outstanding hemocompatibility suitable for intravenous administration in vivo.

### 3.3. Therapeutic Effects of Siv@NMs in Sepsis

**Figure 3:**
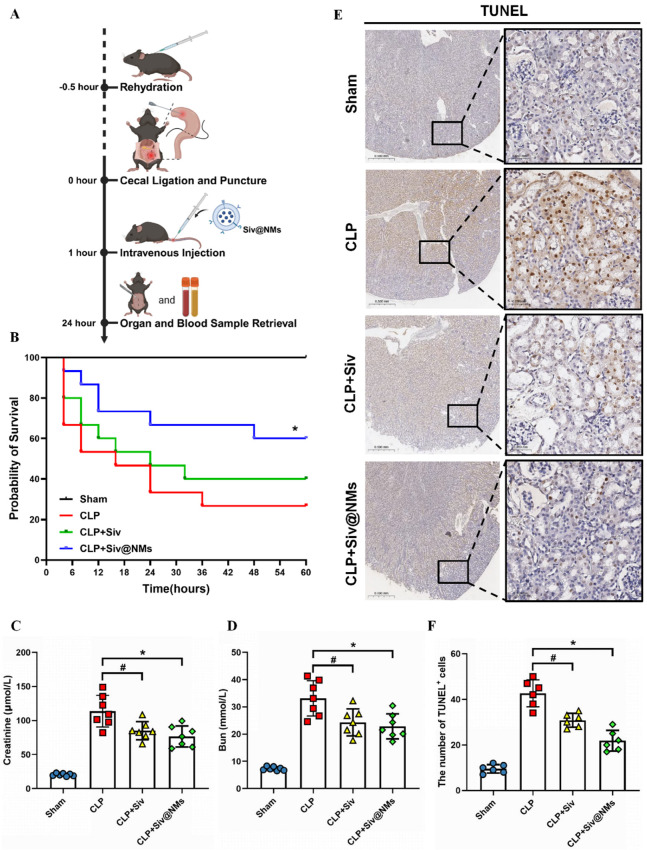
Therapeutic efficacy of Siv@NMs. (**A**) Therapeutic diagram of Siv@NMs in the mouse model of CLP-induced sepsis. (**B**) Kaplan–Meier survival curves illustrating survival rates (Sham group, n = 10; all other groups, n = 15). (**C**,**D**) Creatinine and BUN levels in serum for assessing kidney injury and renal function (n = 6). (**E**) Representative TUNEL staining images at 20× and 40× magnification, illustrating apoptotic cells in kidneys. Insets highlight areas with TUNEL-positive cells. (**F**) Quantification of TUNEL-positive cells, indicating apoptosis levels in kidney tissues (n = 5). Data are shown as the means ± SD; ^#^ *p* < 0.05, CLP+Siv vs. CLP group; * *p* < 0.05, CLP+Siv@NMs vs. CLP group.

A therapeutic diagram of Siv@NMs in the mouse model of CLP-induced sepsis is shown in Figure 3A. The Kaplan–Meier survival analysis demonstrated distinct therapeutic outcomes among treatment groups. While free sivelestat (CLP+Siv) conferred a moderate survival benefit compared to the CLP-only group, Siv@NMs (CLP+Siv@NMs) treatment resulted in significantly enhanced survival rates, highlighting the superior therapeutic potential of the nanoparticle formulation in sepsis management (Figure 3B). Renal function assessment revealed significant pathological changes in CLP-induced sepsis. Both serum creatinine and blood urea nitrogen (BUN) levels were markedly elevated in the CLP group compared to Sham controls, indicating severe kidney injury (Figure 3C,D). Although free sivelestat partially attenuated these elevations, Siv@NMs treatment showed significantly greater reductions in both renal biomarkers, demonstrating enhanced renoprotective effects of this formulation under septic conditions (Figure 3C,D).

In addition, TUNEL staining was performed on kidney sections to evaluate cell apoptosis. The CLP group exhibited a substantial increase in TUNEL-positive cells compared with the Sham group, reflecting widespread apoptotic damage. While treatment with free sivelestat moderately reduced apoptosis, Siv@NMs administration resulted in the most significant reduction in TUNEL-positive cells among all septic groups. These findings suggest that Siv@NMs possess superior anti-apoptotic properties that effectively preserve renal cellular integrity during sepsis (Figure 3E,F).

### 3.4. Therapeutic Effects of Siv@NMs on Renal NET Formation in Septic Conditions

**Figure 4:**
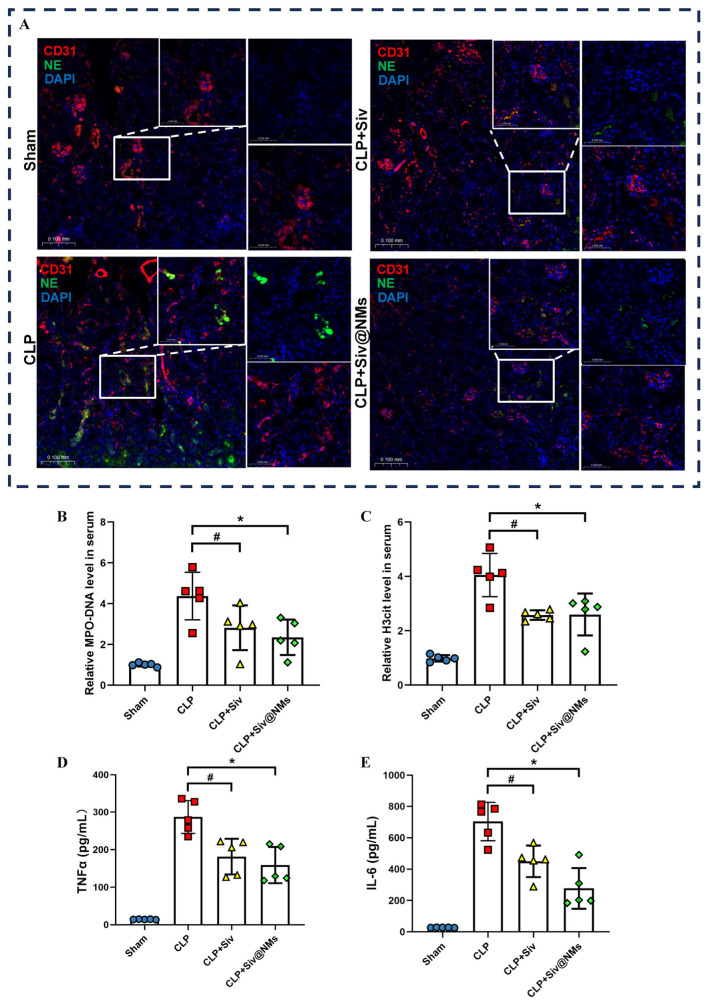
Therapeutic effects of Siv@NMs on renal NET formation in septic conditions. (**A**) Immunofluorescent staining of renal tissues. Staining shows CD31 (red), NE (green), and DAPI (blue) to assess endothelial injury and NET formation. Immunofluorescent co-localization was performed to assess NET formation (NE) in endothelial injury. (**B**,**C**) Relative expression of serum NET biomarkers MPO-DNA and H3cit measured by ELISA (n = 5). (**D**,**E**) Relative expression of serum inflammatory cytokines TNFα and IL-6. Data are shown as the means ± SD; ^#^ *p* < 0.05, CLP+Siv vs. CLP group; * *p* < 0.05, CLP+Siv@NMs vs. CLP group.

Immunofluorescence analysis of kidney sections revealed distinct patterns of neutrophil extracellular trap (NET) formation across experimental groups. Vascular endothelium, labeled with CD31 (red), showed co-localization with neutrophil elastase (NE, green) signals, indicating NET deposition (Figure 4A). Compared to the Sham group, the CLP group exhibited intense NE fluorescence, reflecting substantial NET formation in septic conditions. Treatment with free sivelestat resulted in a moderate reduction in NE signals, whereas Siv@NMs led to a dramatic decrease in NET-associated fluorescence, suggesting superior inhibitory effects on pathological NET formation (Figure 4A).

Quantitative assessment of circulating NET biomarkers confirmed these observations. Serum levels of both MPO-DNA complexes and citrullinated histone H3 (H3cit) were significantly elevated in the CLP group compared to Sham controls. Free sivelestat treatment partially attenuated these elevations (Figure 4B–E). Notably, Siv@NMs treatment achieved significantly greater reductions in MPO-DNA, H3cit, TNFα, and IL-6 levels, further validating the enhanced therapeutic efficacy of the nanoparticle formulation in suppressing systemic NETosis and inflammation during sepsis.

### 3.5. Therapeutic Effects of Siv@NMs on Endothelial Dysfunction

**Figure 5:**
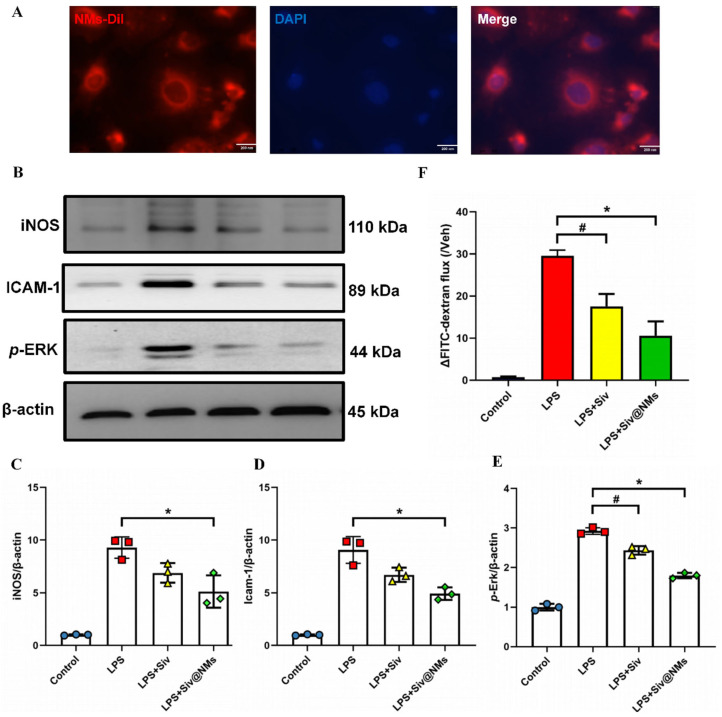
Therapeutic effects of Siv@NMs on endothelial dysfunction. (**A**) Co-localization of Dil-labeled NMs (red) and DAPI-stained HUVEC nuclei (blue) following incubation incubated with HUVECs. Scale bar =10 μm. (**B**) Western blotting was performed to detect iNOS, ICAM-1, and *p*-ERK expression. (**C**–**E**) Quantification of protein band intensities normalized to GADPH, presented as fold changes relative to the control group. (**F**) FITC-dextran assay. ^#^ *p* < 0.05, LPS+Siv vs. LPS group; * *p* < 0.05, LPS+Siv@NMs vs. LPS group.

Confocal fluorescence microscopy analysis demonstrated efficient cellular uptake of Dil-labeled Siv@NMs (red) by HUVECs, as evidenced by co-localization with DAPI-labeled nuclei (blue; Figure 5A). This robust internalization suggests a direct mechanism of action through endothelial cell engagement to counteract LPS-induced dysfunction. Moreover, Western blot analysis revealed significant LPS-induced upregulation of inflammatory mediators, with iNOS, ICAM-1, and *p*-ERK protein levels in HUVECs. Although free sivelestat partially attenuated these elevations, Siv@NMs treatment achieved more substantial suppression of iNOS, ICAM-1, and *p*-ERK, indicating superior anti-inflammatory efficacy at the molecular level (Figure 5B–E). Furthermore, endothelial barrier integrity was quantitatively assessed using FITC-dextran permeability assays. The LPS-treated group exhibited a pronounced increase in dextran flux, signifying a compromised endothelial monolayer. Siv@NMs treatment showed significantly greater protection compared with free sivelestat, maintaining barrier function at near-normal levels, highlighting their excellent capacity to preserve endothelial integrity under LPS challenge.

### 3.6. Therapeutic Effects of Siv@NMs on Mitochondrial Dysfunction and Oxidative Stress in Endothelial Cells

**Figure 6:**
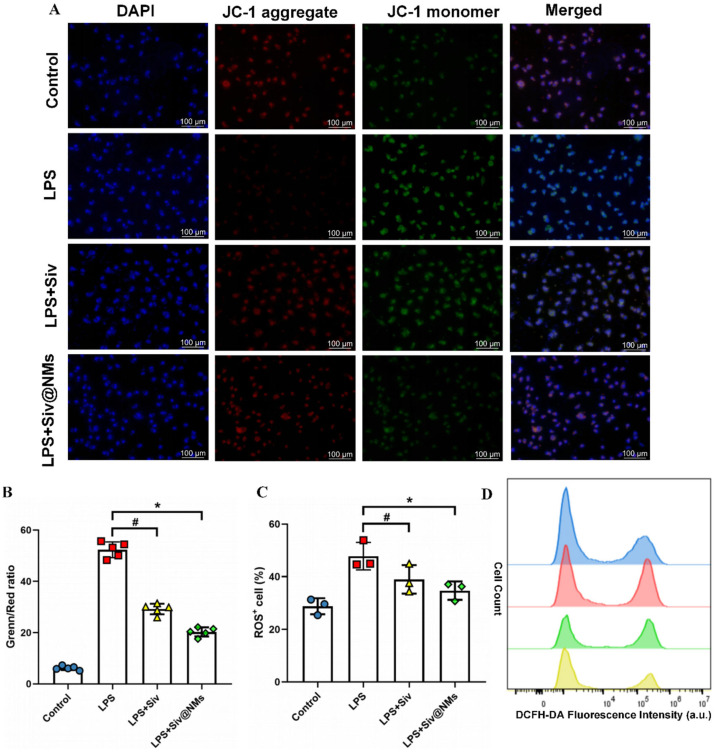
Therapeutic effects of Siv@NMs on mitochondrial dysfunction and oxidative stress in endothelial cells. (**A**) Representative fluorescence microscopy images of JC-1 staining. (**B**) Quantitative analysis of JC-1 fluorescence ratio (green/red) using a microplate reader (n = 5). (**C**,**D**) Percentage of ROS^+^ cells quantified and representative flow cytometry histograms by FCM. ^#^ *p* < 0.05, LPS+Siv vs. LPS group; * *p* < 0.05, LPS+Siv@NMs vs. LPS group.

JC-1 staining analysis illustrated significant LPS-induced mitochondrial dysfunction in endothelial cells, characterized by a marked reduction in red fluorescence (JC-1 aggregates) and concomitant increase in green fluorescence (JC-1 monomers), indicating profound mitochondrial membrane depolarization. Our results showed that Siv@NMs exhibited more pronounced mitochondrial protective effects compared with the free sivelestat group (Figure 6A,B). In addition, quantitative flow cytometry using DCFH-DA evaluated the impact of Siv@NMs on intracellular ROS production. Data demonstrated that LPS stimulation induced an obvious increase in ROS-positive cells compared to controls. Free sivelestat partially mitigated ROS production, whereas Siv@NMs treatment resulted in significantly greater ROS suppression. The corresponding histograms further confirmed these findings, displaying a leftward shift in fluorescence intensity that suggested the enhanced ability of Siv@NMs to alleviate oxidative stress in LPS-stimulated endothelial cells.

## 4. Discussion

Our study demonstrated that targeted delivery of sivelestat to injured endothelium via neutrophil-membrane-coated nanoparticles (Siv@NMs) substantially improved therapeutic outcomes in sepsis. Through isolation of neutrophil membranes and successful encapsulation of sivelestat, we developed Siv@NMs that retain the biological activity of neutrophil membranes, establishing an effective platform for endothelial targeting and enhanced therapeutic efficacy.

Current treatment protocols for sepsis or ARDS suggest intravenous sivelestat administered twice daily at 12 h intervals as an alternative to approved regimens. However, septic patients typically require continuous sivelestat infusion for up to two weeks to achieve the optimal therapeutic benefit [13]. In contrast, Siv@NMs demonstrated significantly improved survival rates in septic animal models compared to free sivelestat. Survival analyses revealed that Siv@NMs provide substantial protective effects during sepsis’ early phase, suggesting that neutrophil membrane encapsulation not only enhances sivelestat’s therapeutic efficacy but may also reduce the required treatment duration. We acknowledge that evidence on the individual contributions of key neutrophil integrins to endothelial targeting remains limited, and we are addressing this in ongoing follow-up experiments using optimized integrin-blocking and gene-silencing approaches. Collectively, our findings indicate that neutrophil-membrane-mediated targeted delivery optimizes the pharmacological profile of sivelestat, potentially reducing both the dosage and treatment period for managing sepsis-induced organ dysfunction.

Organs, including the kidney [32], small intestinal microvessels, and lung [33], represent common sites for sepsis-induced NETs formation, characterized by high vascular endothelial cell concentrations. Given the critical role of the kidney in sepsis-associated organ dysfunction and its abundant endothelial content [34], we selected it for detailed observation. Our findings showed that treatment with Siv@NMs effectively attenuated renal dysfunction while reducing NET formation, demonstrating the potential of targeted sivelestat delivery in protecting endothelial integrity and mitigating organ injury. During sepsis, the compromised endothelial glycocalyx exposes adhesion molecules and antigens that promote leukocyte chemotaxis [35]. As the most sensitive leukocyte subpopulation, neutrophils rapidly adhere to and interact with the damaged endothelium [36,37]. Notably, the neutrophil membrane incorporated into nanoparticles shows particular promise for targeted delivery, primarily through β-integrin-mediated adhesion to activated endothelial cells, which facilitates site-specific accumulation [38,39].

Building on these pathophysiological insights, emerging cell membrane coating technologies now enable nanoparticle functionalization for specific microenvironments [40]. By leveraging sepsis-associated physiological alterations, we encapsulated nanomedicines within neutrophil membranes to achieve targeted delivery to compromised vasculature, potentially enhancing therapeutic efficacy. Immunofluorescence co-localization experiments confirmed efficient HUVEC internalization of Siv@NMs. In vitro studies using HUVECs employed lower sivelestat concentrations [41], with FITC-dextran permeability assays showing that while free sivelestat at reduced doses provided some endothelial barrier protection, Siv@NMs delivered significantly superior protective effects. Moreover, Western blot analysis further demonstrated a marked attenuation of endothelial inflammatory responses and adhesion molecule expression, underscoring Siv@NMs’ enhanced efficacy in preserving endothelial function during sepsis.

Furthermore, the formulation preserved mitochondrial function and maintained endothelial barrier integrity under LPS-induced stress. Excessive ROS and RNS production impair mitochondrial function, leading to endothelial injury and cell death [42,43]. Inhibiting free radical generation to protect endothelial cells remains crucial, with ROS-induced endothelial dysfunction prevention representing a longstanding therapeutic strategy in sepsis [44,45]. Traditional treatments emphasize early fluid resuscitation, vasopressor support, and prompt broad-spectrum antibiotic administration [46,47,48]. However, previous approaches targeting NO synthesis inhibition or ROS elimination have failed clinically, likely due to insufficient neutralization of active free radicals and poor targeting of drugs to endothelial cells [49]. In our study, Siv@NMs effectively suppressed iNOS expression in endothelial cells, with ROS level and mitochondrial membrane potential assessments confirming significant ROS reduction. Furthermore, neutrophil membrane-encapsulated formulations specifically target inflamed tissues, thereby enhancing drug bioavailability and reducing adverse effects, and evading immune clearance. Considering the central role of mitochondria in cellular energy metabolism and apoptotic pathway regulation, preserving mitochondrial function proves essential for preventing endothelial cell death and subsequent organ dysfunction during sepsis [50,51]. Mitochondrial dysfunction not only disrupts energy production but also amplifies inflammatory responses through the release of mitochondrial damage-associated molecular patterns (mtDAMPs), exacerbating endothelial injury [52]. Siv@NMs both mitigate oxidative stress and maintain mitochondrial membrane potential, thereby disrupting the inflammation–cellular damage cycle. This mitochondrial preservation likely contributes to improved endothelial homeostasis, emphasizing the potential of Siv@NMs as a targeted therapeutic platform for addressing sepsis-induced organ failure.

In summary, our research illustrated the preparation and therapeutic mechanism of Siv@NMs for sepsis treatment. By encapsulating sivelestat within neutrophil-derived membranes that retain inherent targeting capabilities, administered Siv@NMs intravenously circulated and preferentially bound to inflamed vascular endothelium in sepsis. Through neutrophil elastase inhibition and NET formation, Siv@NMs protected against endothelial dysfunction while mitigating excessive ROS-mediated damage. This targeted delivery system shows significant promise for attenuating organ injury and improving clinical outcomes in sepsis.

## Data Availability

The original contributions presented in this study are included in the article. Further inquiries can be directed to the corresponding authors.
